# Dynamic Modulation of a Learned Motor Skill for Its Recruitment

**DOI:** 10.3389/fncom.2020.457682

**Published:** 2020-12-23

**Authors:** Kyuengbo Min, Jongho Lee, Shinji Kakei

**Affiliations:** ^1^Department of Psychiatry & Behavioral Sciences, Tokyo Metropolitan Institute of Medical Science, Tokyo, Japan; ^2^Department of Clinical Engineering, Faculty of Health Sciences, Komatsu University, Komatsu, Japan

**Keywords:** motor skill recruitment, muscle synergy, corticospinal tract, reinforcement learning, cortico-basal ganglia circuit, muscle loading, feedback gain control

## Abstract

Humans learn motor skills (MSs) through practice and experience and may then retain them for recruitment, which is effective as a rapid response for novel contexts. For an MS to be recruited for novel contexts, its recruitment range must be extended. In addressing this issue, we hypothesized that an MS is dynamically modulated according to the feedback context to expand its recruitment range into novel contexts, which do not involve the learning of an MS. The following two sub-issues are considered. We previously demonstrated that the learned MS could be recruited in novel contexts through its modulation, which is driven by dynamically regulating the synergistic redundancy between muscles according to the feedback context. However, this modulation is trained in the dynamics under the MS learning context. Learning an MS in a specific condition naturally causes movement deviation from the desired state when the MS is executed in a novel context. We hypothesized that this deviation can be reduced with the additional modulation of an MS, which tunes the MS-produced muscle activities by using the feedback gain signals driven by the deviation from the desired state. Based on this hypothesis, we propose a feedback gain signal-driven tuning model of a learned MS for its robust recruitment. This model is based on the neurophysiological architecture in the cortico-basal ganglia circuit, in which an MS is plausibly retained as it was learned and is then recruited by tuning its muscle control signals according to the feedback context. In this study, through computational simulation, we show that the proposed model may be used to neurophysiologically describe the recruitment of a learned MS in novel contexts.

## Introduction

Innate and learned motor skills (MSs) are recruited in the central nervous system (CNS) for effective and fast motor control in response to novel external circumstances such as disturbances. To recruit an MS in response to novel contexts, its contextual information must be afferently transmitted to the CNS through feedback control processes. Therefore, the recruitment of an MS should be considered in the feedback control process. However, this mechanism has not been addressed in previous studies related to feedback control, such as proportional integral derivative control (Petkos and Vijayakunar, 2007) and optimal feedback control (Todorov and Jordan, 2002; Liu and Todorov, 2007), because these studies focused only on correcting motor control errors through feedback gain control. In addressing this issue, we hypothesized that an MS is dynamically modulated according to the feedback context to expand its recruitment range into novel contexts, which do not involve the learning of an MS. The following two sub-issues are taken into account in this article.

Dynamic modulation of an MS in response to the feedback context is a mechanism that allows rapid recruitment of an MS in a novel context. In validating this hypothesis, the Synergy strategy-based muscle Control (SC) proposed in our previous study (Min et al., 2018) is a valuable concept because it contributes to the dynamic modulation of an MS to regulate the functional redundancy of individual muscle units for the feedback context. To achieve this SC-driven MS (SC-MS), all muscle units contributing to an MS need to be classified into multiple group units according to their innervation by peripheral nerves derived from the brachial plexus. Consequently, these group units cause the contracting sets of muscles, termed motor primitives (MPs) (Giszter et al., 1993), to effectively suppress the control redundancy of muscle units in feedback control. In our previous study (Min et al., 2018), this muscle control policy was defined as the group control policy (GCP) that outputs the same control signal to all muscle units constituting a group unit. Although the GCP is an effective control policy for suppressing the control redundancy of muscle units in feedback control, it needs assistance to generate novel patterns of muscle activities that cannot be produced by combining group units. To assist GCP, the individual control policy (ICP) was defined as the control policy for outputting identified individual control signals to individual muscle units. These two control policies synergistically combine to optimally control muscle units according to the feedback context. This synergy may neurophysiologically correspond to the combination of corticospinal neurons (CSTs) in the primary motor cortex (M1) and its second type of CSTs, termed cortico-motoneuronal cells (CMs), through the corticospinal tract, which was addressed in a previous study (Rathelot and Strick, 2009). In this previous study, it was suggested that the MPs activated by the CSTs in M1 through their connection with interneurons in the spinal cord may be adjusted by the signals that are produced from CMs through their monosynaptic connection with motor neurons (MNs) in the spinal cord. This adjusting of MPs may sculpt novel motor output patterns for highly skilled movements that cannot be produced by combining MPs. Consequently, the combination of two ways of controlling muscles in the corticospinal tract is more plausible in neurophysiologically representing an MS than CSTs driven one way, which is the route for controlling MPs termed muscle synergies (Tresch et al., 1999; d'Avella et al., 2003; Torres-Oviedo et al., 2006; Safavynia and Ting, 2012; Ting and Macpherson, 2012; Barroso et al., 2014; Suzuki et al., 2017; Amundsen Huffmaster et al., 2018; De Marchis et al., 2018; Kibushi et al., 2018; Toma and Santello, 2019), whose individual MP units are composed of spatiotemporally fixed muscle activities. These studies demonstrate that SC may be neurophysiologically suitable for characterizing the dynamic modulation of an MS.

Even if an MS is dynamically modulated for its recruitment in novel feedback contexts, this modulation is trained in the dynamics under the MS learning context. Therefore, this learning condition of an MS naturally brings about movement control deviation from the desired state in a novel context. To overcome this handicap, a learned MS needs to be modified in response to a novel context. In addressing this issue, we propose a Tuned Synergy strategy-based muscle Control (T-SC) model, in which the SC-MS is tuned in response to the feedback context. Through this tuning, the aforementioned deviation is supposedly reduced. In designing this model, we assumed that the tuning signals of the SC-MS are cumulatively modified to tune SC-MS-produced muscle activities according to the deviation from the desired movement, which is recognized through feedback control. This hypothesis is based on experimental evidence demonstrating that the response through sensorimotor control is coupled with ongoing decision processes, which are reflected by the accumulated feedback information (Selen et al., 2012). In a previous related study (d'Avella and Pai, 2010), this issue was also addressed with regard to the limited recruitment range of existing modules such as muscle synergies in novel contexts. However, an alternative solution, apart from learning a new MS, has not been suggested so far. The proposed T-SC may be an alternative motion control strategy for novel contexts because it is more efficient for the rapid adaptation of motion control in novel contexts than learning a new MS.

The neurophysiological architecture and mathematical description of the T-SC model are presented in sections Neurophysiological Architecture and Mathematical Model, respectively. To validate this model, we simulated the recruitment of the SC-MS in novel contexts that were not present when the MS was learned (section Results).

## Materials and Methods

### Neurophysiological Architecture

The neurophysiological architecture of T-SC is based on experimental evidence (Spraker et al., 2007) showing that the cortico-basal ganglia (cortico-BG) circuit is involved in scaling the force generation according to the external environment. Accordingly, this evidence is applicable to validating the recruitment of a learned SC-MS through tuning its muscle force control signals according to the feedback context. In this architecture, the operation of T-SC in the CNS may be achieved as follows.

Based on the experimental evidence (Pruszynski et al., 2011) for involvement of the M1 region in modulating the proprioceptive response related to the knowledge of limb mechanics, we surmised that the sensory feedback signals, **s**_fb_, including the contextual information for the dynamic states of the skeletal joint, are transferred to M1 through its somatosensory pathway (London and Miller, 2013). These feedback signals, **s**_fb_, are inputted to the basal ganglia (BG) through M1. In the cortico-BG loop (Barto, 1995; Doya, 2000, 2007, 2008; Ito and Doya, 2011), the BG selectively disinhibits the activities of both M1 and the brainstem to select the optimal tactic for motion control (Hikosaka et al., 2000). The extent of this disinhibition is controlled via dopamine release (Shinnamon, 1993) during reinforcement learning (Houk et al., 1995). Therefore, the BG is assumed to dynamically produce a trade-off between inhibition and disinhibition of the activity in M1 during sequential motion control (Nambu et al., 2002). Further, section Recruiting a Learned MS via the Cortico-Basal Ganglia Loop discusses the neurophysiological evidence for the involvement of the BG in kinematic control through the recruitment of a learned MS. Based on this experimental evidence, in the BG, we assumed that an SC-MS is dynamically modulated by inhibiting or disinhibiting the GCP and ICP, which regulate the functional redundancy of individual muscle units for **s**_fb_, as discussed in section Introduction. This modulation is mathematically described in section Dynamic Modulation of an SC-MS. Through this modulation, the SC-MS produces a muscle control signal, ${\text{u}}^{{{\text{P}}_{t}}^{\text{SC}}}$, that is efferently copied, as **u**^SC^, to the spinal cord through the corticospinal neurons (CSTs) in M1. Through this process, a learned SC-MS retained in the BG is dynamically modulated to produce **u**^SC^ according to the feedback context **s**_fb_. A discussion of this modulation is introduced in sections Recruiting a learned MS via the Cortico-Basal Ganglia Loop and Muscle Control Scheme of the Corticospinal Tract in Recruiting an SC-MS.

The signals **u**^SC^ are assumed to be tuned in M1 because experimental evidence (Herter et al., 2009) has shown that neural activity in M1 is broadly tuned to novel contexts, such as mechanical perturbations applied to the shoulder and elbow, and reflects knowledge of joint–limb dynamics (Pruszynski et al., 2011). Based on this supposition, the **u**^SC^ may be tuned with the following dynamic modulation process for the feedback context. Concurrent with the aforementioned input of **s**_fb_ to M1, the goal states **s**^o^ of **s**_fb_ are also input to M1 from the association cortex. Both **s**_fb_ and **s**^o^ are inputs to the muscle loading tuner and the difference between the two signals is transferred to the tuning gain (TG), **G**^tuning^, of **u**^SC^. The **G**^tuning^ consists of agonist and antagonist loading signals, which disinhibit the activities of loaded muscles and inhibit the activities of unloaded muscles by properly scaling them (Nashed et al., 2015). This tuning process generates the optimal muscle control signals u^*^, which descend to MNs in the spinal cord to control the muscles. This tuning is mathematically described in section Tuning of a Learned SC-MS.

### Mathematical Model

#### Dynamic Modulation of an SC-MS

As mentioned in section Introduction, we have defined the group units as muscle control units, which produce the contracting sets of muscles, termed MPs. These group units and their belongings are determined according to the peripheral nerves innervating them (Table 1). Based on this neurophysiological definition, the GCP is defined as the control policy considering individual muscle units as a component of the group unit, in which all components respond to the feedback context with one common signal. In contrast to the GCP, the ICP is defined as the control policy considering individual muscle units as independent units of the group units, thereby controlling individual muscle units with their identified signals. Therefore, by optimizing the synergy between these two control policies for a feedback context, an SC-MS is dynamically modulated for the feedback context. This modulation is mathematically defined by the following model based on our previous study (Min et al., 2018):

(1) $$\begin{array}{l} {\text{P}}_{t}^{\text{SC}}=\left(\nu^{\text{G}},\nu^{\text{I}}\right),\nu^{\text{G}}=\sigma \left({\text{s}}^{t}\right)=\exp\left(-0.5V\left({\text{s}}^{t}\right)\right),\;\nu^{\text{I}}=1-\nu^{\text{G}} \end{array}$$

where **P**_*t*_^SC^ is the synergy coordinate of the GCP weight *v*^G^ and ICP weight *v*^I^. This is determined by the critic value (CV) V(**s**^*t*^), which evaluates the potential of the feedback contextual vector **s**^*t*^ at time *t* for reaching the goal state. As the **s**^*t*^ is produced through the performance of the SC-MS, the V(**s**^*t*^) presents an evaluation of the performance of the SC-MS for the goal state. Therefore, the **P**_*t*_^SC^ is dynamically optimized according to the performance of the SC-MS at time *t* for the goal state. The synergy between *v*^G^ and *v*^I^ is simulated in Figures 4, 5A, 6A, 7A (see section Results). By applying the **P**_*t*_^SC^ to Equation (2b), the SC-MS, using the actor model in Equation (2a), is dynamically modulated. This achievement is rewarded by functionally improving the V(**s**^*t*^) (Min et al., 2018). Consequently, this improvement reinforces the SC-MS to achieve its goal state. This CV-driven reinforcement learning is based on the actor–critic model (Barto, 1995; Sutton and Barto, 1998), which is designed to simulate reinforcement learning (Houk et al., 1995) in the BG. The simulation condition of this learning is precisely described in section Learning and Recruitment Condition of an SC-MS.

**Table 1:** Elements of the neuromuscular system controlling the elbow joint.

**Flexors**	
Group 1 (radial nerve)	Brachioradialis
Group 2 (musculocutaneous nerve)	Biceps brachii (long head), Biceps brachii (short head), and brachialis
Group 3 (median nerve)	Pronator teres
**Extensors**	
Group 4 (radial nerve)	Triceps brachii (lateral head), Triceps brachii (long head), Triceps brachii (medial head), and anconeus

Using **P**_*t*_^SC^ optimized through the aforementioned learning, the SC-MS is dynamically modulated to generate the muscle control signals **u**^SC^ as follows:

(2a) $$\begin{array}{l} {U_{i}}^{\text{SC}}\left({\text{s}}^{t}\right)≅u_{i}\left({\text{s}}^{t};\text{W}\right)\\ \;={u_{i}}^{\max}\text{sig}\left(\overset{K}{\underset{k=1}{\sum }}{W_{k}}^{i}b_{k}\left({\text{s}}^{t}\right)+\sigma \left({\text{s}}^{t}\right)n_{i}\left(t\right)-\text{B}\right),\\ \;{u_{i}}^{\max}=1.0,\;\sigma \left({\text{s}}^{t}\right)=\sigma_{0}\exp\left(-0.5V\left({\text{s}}^{t}\right)\right),\\ \;b_{k}\left({\text{s}}^{t}\right)=\frac{A_{k}\left({\text{s}}^{\;t}\right)}{\overset{K}{\underset{l=1}{\sum }}A_{l}\left({\text{s}}^{\;t}\right)}\;,\;A_{k}\left({\text{s}}^{t}\right)=\exp\left[-\overset{n}{\underset{i=1}{\sum }}{\left(\frac{{s_{i}}^{t}-{c_{i}}^{k}}{{\sigma_{i}}^{k}}\right)}^{2}\right]\; \end{array}$$

(2b) $$W_{k}^{\;i}=v^{\text{I}}w_{k}^{\;i}+\nu^{\text{G}}w_{k}^{\;g}$$

where the U_*i*_^SC^(**s**^*t*^) functions as the actor generating the control signal of the *i*th muscle of **u**^SC^ and **sig**(*x*) is the sigmoid function. The function n_*i*_(*t*) produces the white noise in determining the activities of individual muscles. The magnitude of n_*i*_(*t*) is determined according to σ(**s**^*t*^) by considering V(**s**^*t*^). σ_0_ is a constant parameter. This noise is designed to enhance the learning dynamic of an MS, thereby being suppressed by setting σ_0_ to zero in simulating its recruitment. B is the parameter controlling the baseline of **sig**(*x*), i.e., the value of **sig**(*x* = 0.0). The base function b_*k*_(**s**^*t*^) is the *k*th element of a normalized Gaussian network (NGSN). *K* is the total number of base functions. The node of b_*k*_(**s**^*t*^) is defined as the parameter $c_{i}^{\;k}$, which is the *i*th element of the center of b_*k*_(**s**^*t*^) and $\sigma_{i}^{\;k}$ is its range. This $c_{i}^{\;k}$ is determined before the learning takes place. As the state vector **s**^*t*^ comprises the joint angle and its velocity, the predetermined format of the NGSN is designed based on the grid distribution of the two-dimensional state by setting the total number of state elements *n* to 2. The symbol $s_{i}^{\;t}$ is the *i*th element of the contextual vector **s**^*t*^. $W_{k}^{\;i}$ is the network weight of b_k_(**s**^*t*^) in producing the *i*th muscle activity. As described in Equation (2b), the $W_{k}^{\;i}$ is the summation of $w_{k}^{\;i}$ and *w*_*k*_^g^, which are, respectively, weighted by *v*^I^ and *v*^G^ of **P**_*t*_^*SC*^. The parameter *w*_*k*_^*g*^ is the *k*th NGSN weight of the *g*th group unit, which is governed by the GCP, whereas *w*_*k*_^*i*^ is the *k*th NGSN weight of the *i*th muscle affiliated to the *g*th group unit, which is governed by the ICP. The weights *w*_*k*_^*i*^ and *w*_*k*_^*g*^ are optimized through the aforementioned SC-MS learning. For further information, including the optimizing process of *w*_*k*_^*i*^ and *w*_*k*_^*g*^ regarding Equation (2b), refer to our previous study (Min et al., 2018).

#### Tuning of a Learned SC-MS

**Figure 1:**
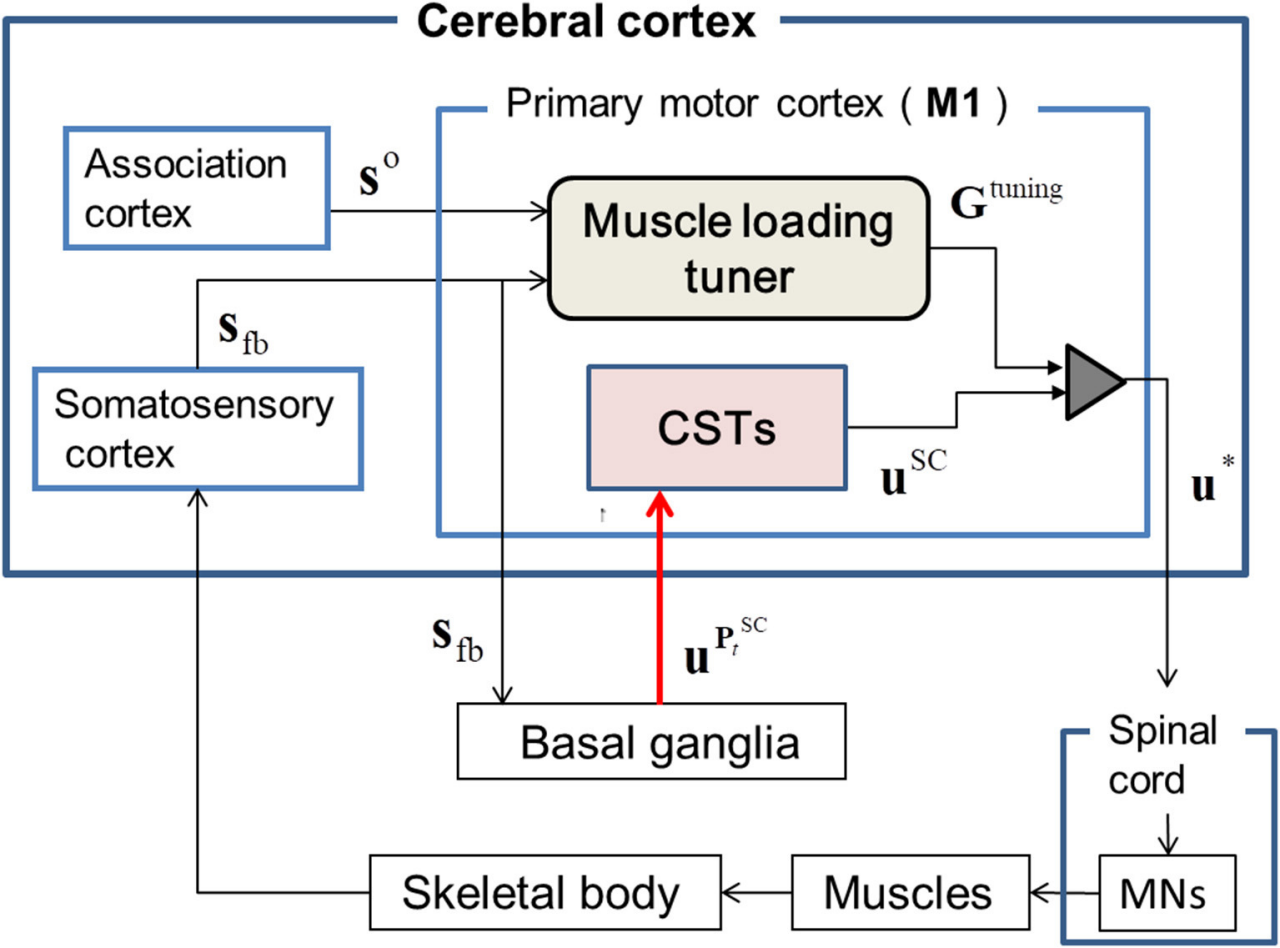
The proposed architecture of the tuned synergy strategy-based muscle control (T-SC). The feedback state **s**_fb_ is transferred to the primary cortex M1 through the transcortical pathway, and the goal state **s**^o^ is transferred to M1 from the association cortex. The basal ganglia estimate the SC signal ${\text{P}}_{\text{t}}^{\;SC}$ that regulates the functional redundancy of individual muscles within the SC for the feedback context **s**_fb_ and outputs ${\text{P}}_{\text{t}}^{\;SC}$ into the corticospinal neurons (CSTs) in M1. The CSTs encode ${\text{P}}_{\text{t}}^{\;SC}$ to **u**^SC^, which are then tuned to u* by the tuning gain signal **G**^tuning^ from the muscle loading tuner. The tuned signals u* are transferred to the skeletal muscles via the spinal cord and the motor neurons (MNs).

As shown in Figure 1, the learned SC-MS-produced **u**^SC^ is additionally tuned to u^*^ with the TG signal **G**^tuning^ from the muscle loading tuner, which is cumulatively modified as the feedback gain parameters of **u**^SC^ according to the deviation from the desired state recognized through feedback control. This tuning is mathematically modeled as follows:

(3) $$\begin{array}{l} {\text{u}}^{*}={\text{G}}^{\text{tuning}}{\text{u}}^{\text{SC}}\text{,}{\text{G}}^{\text{tuning}}=diag\left(G_{\text{0}}^{\text{tuning}},\ldots ,G_{n-1}^{\text{tuning}}\right)\;,\\ {\text{u}}^{\text{SC}}=\left[u_{\text{0}},\ldots ,u_{\text{n}-\text{1}}\right]{\;}^{\text{T}},\text{If }u_{i}\text{ is a flexor, }G_{\text{i}}^{\text{tuning}}=G^{\text{F}}.\\ \text{If }u_{i}\text{ is an extensor, }G_{\text{i}}^{\text{tuning}}=G^{\text{E}}.,\text{i}=0,\ldots ,n-1. \end{array}$$

where **G**^tuning^ functions as a feedback gain parameter that is the diagonal matrix composed of G_*i*_^tuning^. The symbol *n* represents the total number of muscles involved in the motion control. The flexor gain *G*^F^ or the extensor gain *G*^E^ is determined by G_*i*_^tuning^ according to the function of the individual muscles *u*^i^ in controlling the joint.

The **G**^tuning^ is modified by its incremental signal **ΔG**^tuning^ as follows:

(4) $$\begin{array}{l} \Delta {\text{G}}^{\text{tuning}}\left(t\right)=\left[\begin{array}{c} \Delta G^{\text{F}}\left(t\right),\Delta G^{\text{E}}\left(t\right) \end{array}\right]{\;}^{\text{T}}=\text{k}\left(t\right)\cdot \Delta \text{s}\left(t\right),\\ \;\text{k}\left(t\right)=\left[\begin{array}{l} {\text{k}}^{\text{F}}\left(t\right)\\ {\text{k}}^{\text{E}}\left(t\right) \end{array}\right]=\left[\begin{array}{l} k_{\text{p}}(t)\;k_{\text{d}}(t)\;k_{\text{a}}\left(t\right)\\ -k_{\text{p}}\left(t\right)-k_{\text{d}}(t)\;k_{\text{a}}\left(t\right) \end{array}\right],\\ \;\Delta \text{s}\left(t\right)={\text{s}}^{\text{G}}-\text{s}\left(t\right)=\left[\begin{array}{c} \Delta \Theta_{t}^{\text{G}},\Delta {\dot{\Theta }}_{t}^{\text{G}},\Delta {\ddot{\Theta }}_{t}^{\text{G}} \end{array}\right]{\;}^{\text{T}},\\ \;\Delta {\Theta_{t}}^{\text{G}}=\Theta^{G}-\Theta_{t},\Delta {\dot{\Theta }}_{t}^{\text{G}}={\dot{\Theta }}^{G}-{\dot{\Theta }}_{t},\Delta {\ddot{\Theta }}_{t}^{\text{G}}={\ddot{\Theta }}^{G}-{\ddot{\Theta }}_{t} \end{array}$$

where Δ**G**^tuning^(*t*) is composed of the flexor and extensor components, Δ*G*^F^(*t*), Δ*G*^E^(*t*). These two components are estimated by Δ**s**(*t*) and its gain matrix **k**(*t*). Δ**s**(*t*) is the difference between the feedback state $\text{s}\left(t\right)=\left(\Theta_{t},{\dot{\Theta }}_{t},{\ddot{\Theta }}_{t}\right)$ and its desired state ${\text{s}}^{\text{G}}=\left(\Theta^{\text{G}},{\dot{\Theta }}^{\text{G}},{\ddot{\Theta }}^{\text{G}}\right)$, in which both ${\dot{\Theta }}^{\text{G}}$ and ${\ddot{\Theta }}^{\text{G}}$ are zero.

**Figure 2:**
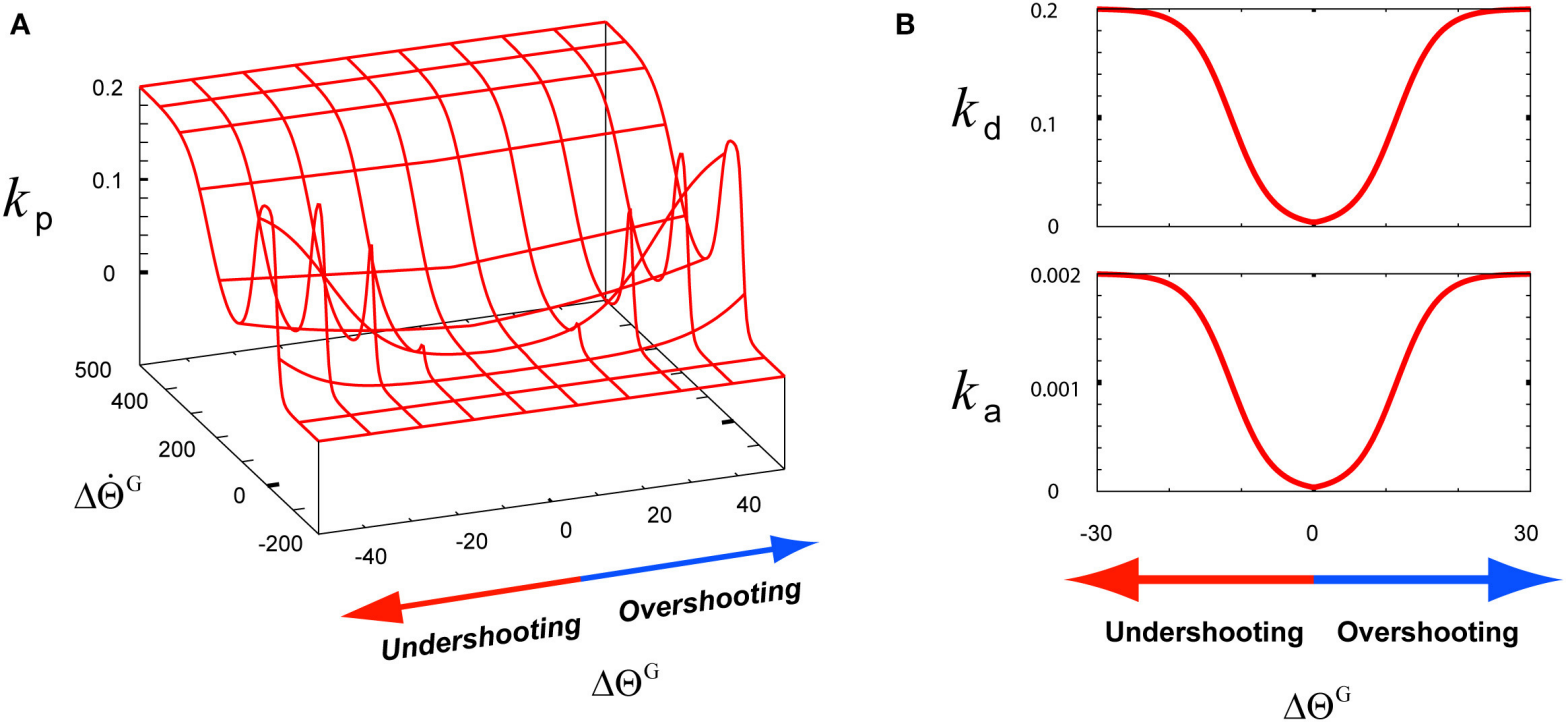
The functions of three k-terms involving the tuning of a motor skill for its recruitment according to the feedback context (ΔΘ^G^, $\Delta {\dot{\Theta }}^{\text{G}}$). **(A)** The function of the angle term *k*_p_. **(B)** The functions of the angular velocity term *k*_d_ and angular acceleration term *k*_a_.

The matrix **k**(*t*) is composed of the following three components: the angle term *k*_p_(*t*), the angular velocity term *k*_d_(*t*), and the angular acceleration term *k*_a_(*t*). These terms contribute to the flexor part ${\text{k}}^{\text{F}}\left(t\right)=\left(k_{\text{p}}\left(t\right),k_{\text{d}}\left(t\right),k_{\text{a}}\left(t\right)\right)$ and the extensor part ${\text{k}}^{\text{E}}\left(t\right)=\left(-k_{\text{p}}\left(t\right),-k_{\text{d}}\left(t\right),k_{\text{a}}\left(t\right)\right)$. The components *k*_p_(*t*) and *k*_d_(*t*) of **k**^E^ are designed as minus terms of **k**^F^ to simulate the activities of extensors. However, the acceleration term *k*_a_(*t*) is set to the same value for both the agonist and the antagonist because the direction of the angular acceleration frequently changes, thus it needs to be suppressed to maintain stable motion control during the co-contraction of both the agonist and the antagonist. These three **k** components are optimally modulated to make the joint angular state approach the goal state by using Equation (5). To achieve this modulation, the three **k** terms of Δ**G**^tuning^(*t*) are modeled to be proportional to $\left||\Delta {\Theta_{t}}^{\text{G}}|\right|$ using the function **sig**(*x*) as follows:

(5) $$\begin{array}{l} k_{\text{p}}(t)={\text{C}}^{k_{\text{p}}}\cdot \text{sig}(\text{ 1.5}\cdot (\Delta {\dot{\Theta }}_{t}^{\text{G}}+(\text{A}\cdot \exp(-\left\|\Delta {\dot{\Theta }}_{t}^{\text{G}}\right\|))\cdot \left\|\Delta {\Theta_{t}}^{\text{G}}\right\|\;\\ \;-\text{B) })\\ k_{\text{d}}(t)={\text{C}}^{k_{\text{d}}}\cdot \text{sig}(\text{D}\left\|\Delta {\Theta_{t}}^{\text{G}}\right\|-\text{B}),\\ k_{a}(t)={\text{C}}^{k_{\text{a}}}\cdot \text{sig}(\text{D}\left\|\Delta {\Theta_{t}}^{\text{G}}\right\|-\text{B}) \end{array}$$

where B (B = 0.4) is the parameter controlling the baseline of **sig**(*x*), that is, the value for **sig**(*x* = 0.0), whereas the parameters C^*k*p^ (C^*k*p^ = 0.2), C^*k*d^ (C^*k*d^ = 0.2), and C^*k*a^ (C^*k*a^ = 0.002) are the constant values of **sig**(*x*). The parameter A (A = 10.0) is the constant gain of the Gaussian function for modulating *k*_p_(*t*), and the parameter D (D = 20.0) is the constant value for modulating *k*_d_(*t*) and *k*_a_(*t*). These *k* components are dynamically modulated considering Δ**s**(t), which was described in Equation (4). As shown in Figure 2A, to model the gain term of $\Delta {{\dot{\Theta }}_{t}}^{\text{G}}$, *k*_p_(*t*) is modeled to mainly function as ${\text{C}}^{k_{\text{p}}}\cdot \text{sig}\left(1.5\cdot \left(\text{A}||\Delta {\Theta_{t}}^{\;\text{G}}||-\text{B}\right)\right)$, which is the sigmoid function of $\Delta {{\dot{\Theta }}_{t}}^{\;\text{G}}$ in the first term of **sig**(). In low-speed undershooting or overshooting, *k*_p_(*t*) functions as ${\text{C}}^{k_{\text{p}}}\cdot \text{sig}\left(1.5\cdot \left(\text{A}||\Delta {\Theta_{t}}^{\;\text{G}}||-\text{B}\right)\right)$, which is the sigmoid function of $\left||\Delta {\Theta_{t}}^{\;\text{G}}|\right|$ in the second term of **sig**(). Using the hybridization of these two terms, *k*_p_(*t*) is modeled as shown in Figure 2A. Due to this modeling, *k*_p_(*t*) responds to $\Delta {{\dot{\Theta }}_{t}}^{\;\text{G}}$ under consideration of $\left||\Delta {\Theta_{t}}^{\;\text{G}}|\right|$. To optimally modulate *k*_d_(*t*) and *k*_a_(*t*) as the gain terms of $\Delta {{\dot{\Theta }}_{t}}^{\;\text{G}}$ and $\Delta {{\ddot{\Theta }}_{t}}^{G}$, their corresponding $\left||\Delta {\Theta_{t}}^{\text{G}}|\right|$ needs to be considered as the feedback context responding to undershooting and overshooting, as shown in Figure 2B. Owing to this consideration, the response of these *k* terms to the feedback context is slower than *k*_p_(*t*) considering $\Delta {{\dot{\Theta }}_{t}}^{\text{G}}$ in high-speed undershooting.

The TG increments generated according to the aforementioned calculations in Equations (4) and (5) are accumulated to modify the corresponding TG signals as follows:

(6) $$\begin{array}{l} {\text{G}}^{\text{tuning}}\left(t\right)={\text{G}}^{\text{tuning}}\left(t-\Delta t\right)+\Delta {\text{G}}^{\text{tuning}}\left(t\right)\text{,}\\ {\text{G}}^{\text{tuning}}\left(t\right)=\left(\begin{array}{c} G^{\text{F}}\left(t\right),G^{\text{E}}\left(t\right) \end{array}\right)\text{,}{\text{ G}}^{\text{tuning}}\left(0\right)=\left(\begin{array}{c} 1.0,1.0 \end{array}\right)\text{.}\\ \text{While }G^{\text{F}}\left(t\right)>0.0,\;\Delta G^{\text{F}}\left(t\right)\text{ is available .}\\ \text{While }G^{\text{E}}\left(t\right)>0.0,\;\Delta G^{\text{E}}\left(t\right)\text{ is available.} \end{array}$$

where the initial TG, **G**^tuning^(0), is set to 1.0 to simulate non-interference by the TG. To achieve this modification, *G*^F^(*t*) and *G*^E^(*t*), termed the flexor and extensor components of **G**^tuning^(*t*), respectively, must be above zero. Therefore, if *G*^F^(*t*) or *G*^E^(*t*) is modified to be below zero, the corresponding signal is set to zero by suppressing its increment.

### Simulation Architecture

**Figure 3:**
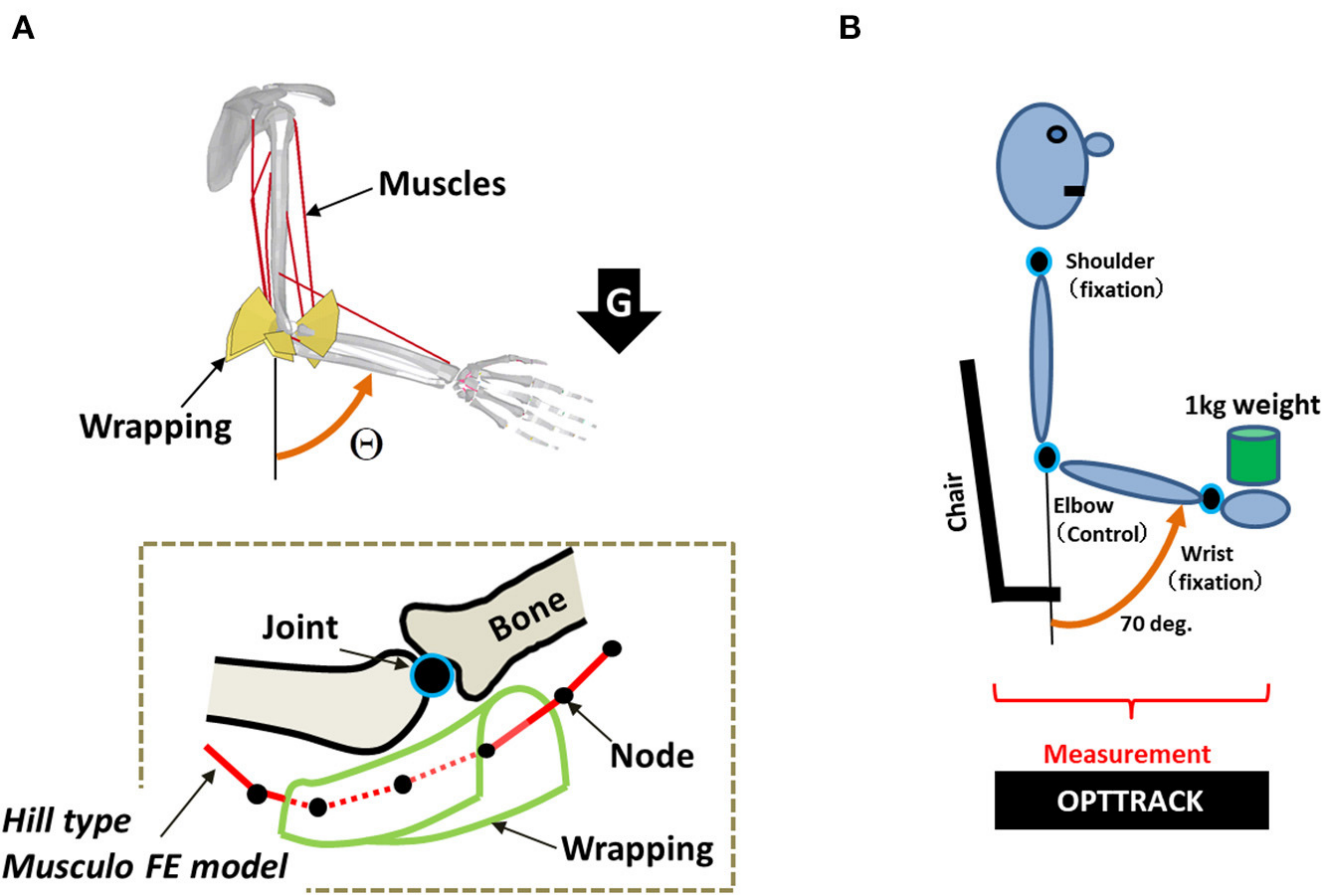
**(A)** A musculoskeletal finite-element (FE) model of the human arm. Each muscle consists of multiple nodes that are used to precisely model its path along with the wrapping. The wrapping is used to keep the path of the muscles within the precise moment arm. **(B)** Experimental setup of human subjects for evaluating the simulation result.

The simulation architecture has been described in detail in our previous study (Min et al., 2018). This architecture is composed of the musculoskeletal finite-element (FE) model (Figure 3), its motion control agent model, and the interface model, which integrates both of the aforementioned models. Precise descriptions are as follows.

#### Musculoskeletal Finite-Element Model

The musculoskeletal model proposed in our previous study (Min et al., 2018) was used for simulating the motion control of a musculoskeletal system. This model was designed using LS-DYNA (Livermore Software Technology Corporation, Livermore, CA, USA), which is an explicit FE code developed for dynamic analyses through simulation. To consider the trade-offs between analytical precision and calculation costs in simulating the motion control of the musculoskeletal FE model, the muscles are designed with FE modeling using multiple bar elements of the muscles that formulate muscle paths between the origin and the insertion points in LS-DYNA. The characteristic features of the muscle forces, which change according to the length of the muscle and its contraction velocity, were modeled using a Hill-type model (Hill and Sec, 1938; Zajac et al., 1985; Thelen, 2003). Anatomical references (Neumann, 2002) were used to align the origin and insertion points and the via points, and to represent the appropriate muscle moment arms using the wrapping contacts (Hada et al., 2007). The predicted muscle moment arms were well-validated against data from several experimental studies (Amis et al., 1979; Murray et al., 1995). As shown in Figure 3A, the proposed FE model consists of two rigid body parts: one representing the upper arm and shoulder, and the other representing the lower arm and hand. The two body parts are linked using a joint constraint that represents the ulnar–humeral joint. The mass of the lower arm was 1.7 kg. The principal moments of inertia of the lower arm body were *I*_11_ = 7.66 × 10^−3^ kg m^2^, *I*_22_ = 7.36 × 10^−3^ kg m^2^, and *I*_33_ = 0.34 × 10^−3^ kg m^2^.

#### Integration of the Musculoskeletal Finite-Element Model and Its Motion Control Agent Model

The entire architecture was implemented through software programming, in which the agent model of the SC-MS was programed with C++ code to perform the learning and recruitment of the LS-DYNA-coded musculoskeletal FE model. This performance was achieved through a C++ code interface model, which was programmed to allow the coding difference between the aforementioned two models.

#### Learning and Recruitment Condition of an SC-MS

To validate the recruitment of the SC-MS under novel conditions involving transient and sustained disturbances, the learning condition of the SC-MS was not affected by any external interference as follows.

In the simulation architecture, the agent model reinforced an MS to be dynamically modulated by the SC, described in Equation (1), for controlling the forearm to reach a goal without any disturbances. Through this reinforcement learning, the agent model learned an SC-MS. During this learning process, the control range of the elbow joint was limited to 30–140°. The aim of this task was to move the hand to its goal position, where the elbow joint angle was at 70°, and to maintain this position. The degree of freedom of the joint was 1. The nine muscles listed in Table 1 were activated to control the elbow in the simulation, as shown in Figure 3A. The time step *t* was 0.01 s. If the total learning time in a trial exceeded 2.0 s or if the angle of the elbow joint was out of the defined control range, a new trial was started after randomly changing the initial position. This process was repeated 780 times.

The SC-MS learned through the above process was recruited in the same time steps as the aforementioned learning time steps. Further information has been provided in detail in our previous study (Min et al., 2018).

### Experimental Setup

To evaluate the proposed simulation model by comparison with the actions of four human subjects (four men, 40–44 years old) under the same conditions as those used in the simulation, we measured the loading responses of the study subjects, which is the same task as that in the simulation. All subjects were healthy and did not have any motor disorders. We assumed that these subjects have learned the MS recruited to achieve the aforementioned novel task throughout their whole life because the goal task of the MS described in section Learning and Recruitment Condition of an SC-MS can be achieved naturally by healthy subjects.

As shown in Figure 3B, in this experiment, the elbow joint angle was measured while the subject held a 1 kg load in his hand. To measure the responses to the loading condition through pure feedback control, the subjects were blindfolded with their eyes closed and were not informed about the timing of the loading. In addition, the distance between the initial falling point of the weight and the initial position of the hand was set close to zero. Furthermore, to approximate the novel condition as closely as possible, only data that were recorded during the first trial for each of the four subjects were used. The subject was instructed to try to recover as soon as possible the preloading posture set at 70°. All subjects were instructed to recover and maintain their preloading posture under this loading condition for 2.0 s. The shoulder and wrist joints were fixed during the measurement of the motion of the elbow joint. In this setting, we measured the positions of the shoulder, elbow, and wrist using OPTOTRAK 3020 (Northern Digital, Waterloo, Ontario, Canada), which is a three-dimensional position measurement device. We then used the measured positions of these three joints to calculate the angular movement of the elbow joint. The experimental setup has been described in detail in our previous study (Min et al., 2018).

All subjects provided written informed consent prior to their participation. The protocol was approved by the Tokyo Metropolitan Institute of Medical Science's ethics committees and was conducted in accordance with the ethical standards of the Declaration of Helsinki.

## Results

As mentioned in section Introduction, because of the dynamic modulation of an MS driven by the SC for novel feedback contexts, an SC-MS may be recruited in the CNS as a valuable learned MS. To validate this supposition, we tested the concept of T-SC in a model that tunes the SC-MS by gaining its signals to robustly recruit it in novel feedback contexts.

As shown in Figure 1, the T-SC is neurophysiologically achieved by tuning the SC-MS according to sensory feedback signals, which are generated in response to the context. Therefore, the T-SC may contribute to recruiting the SC-MS in the feedback control process. To validate this recruitment process, the simulation results of the SC-MS recruitment procedure in response to three novel sustained disturbances that did not involve the learning process of the SC are discussed in this section.

### T-SC in a Novel Sustained Disturbance

**Figure 4:**
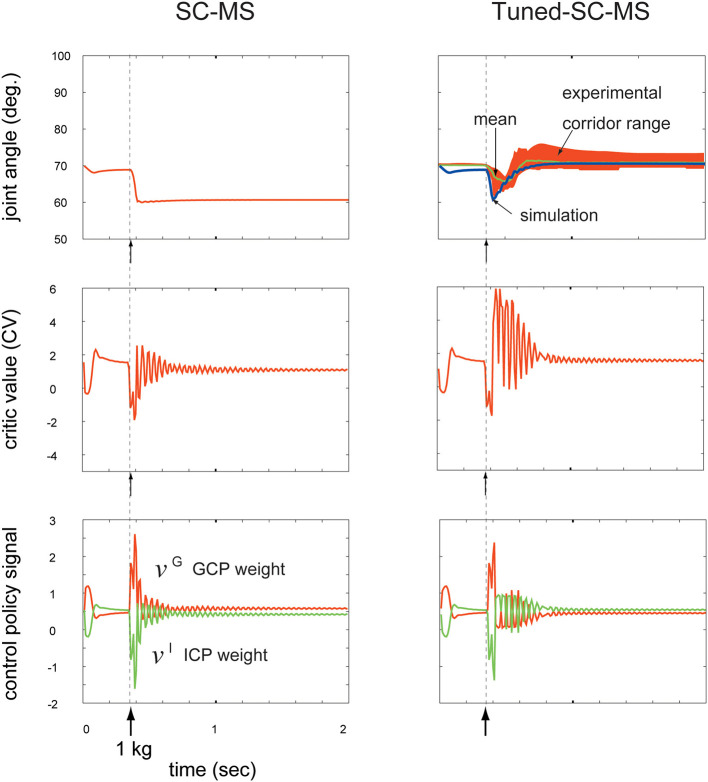
Comparison of a synergy strategy-based muscle control motor skill (SC-MS) and a tuned SC-MS (T-SC-MS) in recruiting the retained motor skill after a 1 kg loading. Top row: Comparison of the SC-MS and T-SC-MS simulations in recruiting the same retained motor skill, and evaluation of the T-SC to reproduce the joint traces experimentally determined in human subjects. Second row: Critic value (CV) according to the joint angular state. Third row: Two control policies according to the CV. GCP, group control policy; ICP, individual control policy.

Novel dynamic contexts in recruiting a learned MS are classified into transient and sustained disturbances. In our previous work (Min et al., 2018), we tested the motion control robustness of the SC-MS in these two types of dynamic contexts that did not involve SC-MS learning. In this test, the SC-MS demonstrated good recruitment against a transient disturbance, such as an impacting force, by recovering the pre-impacted context well. However, the SC-MS revealed the limitations of its recruitment in response to sustained disturbances; it only recovered to the point below the pre-disturbed point, as shown in Figure 4. As this difference in recruiting an SC-MS is attributed to the difference between their loading durations for an SC-MS, we hypothesized that the SC-MS needs to be tuned with accumulative gain signals that consider the duration of the disturbance. To address this issue, we validated the T-SC for novel sustained disturbances as follows.

To validate the T-SC, we compared the performance of the SC-MS and the tuned SC-MS (T-SC-MS) in recovering the preloading posture against a sustained 1 kg loading. This sustained loading did not involve SC-MS learning. The results are shown in the top row of Figure 4. The left column in the top row of Figure 4 demonstrates that the SC-MS found a new posture at 60°, which is below the preloading posture at 70°. By contrast, the T-SC-MS could successfully recover the preloading posture. This simulated recovering joint angular trace was within the real motion trace corridor range derived from the four experimental subjects using the same conditions as the simulation. This achievement of T-SC-MS was simulated through the following T-SC process.

As shown in Figure 1, the joint angular context may be afferently copied to the CNS as the contextual feedback signal and transferred to the BG via M1. According to this feedback signal, striosomal molecules functioning as adaptive critics in the BG (Houk et al., 1995) may estimate the CV as the evaluation of the recruitment of the SC-MS in recovering the preloading state. This is shown in the second row of Figure 4. After sustained 1 kg loading, the CV of the SC-MS decreases accordingly, and remained below, the CV of the preloading state according to maintaining the new posture below the preloading posture after 0.6 s. In comparison with SC-MS recruitment, the CV of the T-SC-MS also decreased during the undershooting, but it recovered to the level of the preloading state. Consequently, the T-SC-MS was more highly valued than the SC-MS in recruiting the learned MS to recover the preloading state after 0.4 s.

According to this CV-based evaluation of the SC-MS recruitment, the BG may optimally regulate the synergistic role redundancy between the GCP and ICP of individual muscles by using the rule based on Equation (1). As mentioned above, the CVs decreased from the preloading value because of their corresponding undershooting, shown in the top row of Figure 4. According to these CVs, as shown in both columns of the third row of Figure 4, the GCP increased whereas the ICP decreased because the GCP-driven group unit control is more effective than the ICP-driven individual muscle unit control in recovering the preloading context during undershooting. This synergy between the GCP and the ICP is differently regulated by SC-MS and T-SC-MS according to their CVs as follows.

After 0.4 s, SC-MS and T-SC-MS differently regulated the synergistic redundancy between the GCP and the ICP compared with before 0.4 s as follows. T-SC-MS started to recover the pre-disturbed CV from its lowest value after 0.4 s. According to this CV recovery, the GCP and ICP started to recover from their highest and lowest values, respectively. After 0.9 s, the two control policies successfully recovered to their pre-disturbed values and were then kept stable at that state. In comparison with the T-SC-MS, the SC-MS maintained the new CV below the pre-disturbed CV after 0.7 s, thereby insufficiently recovering its two pre-disturbed control policies. This comparison is demonstrated in the third row of Figure 4. This superior achievement of T-SC-MS compared with SC-MS for the same novel disturbance is attributed to the following tuning process of an SC-MS.

**Figure 5:**
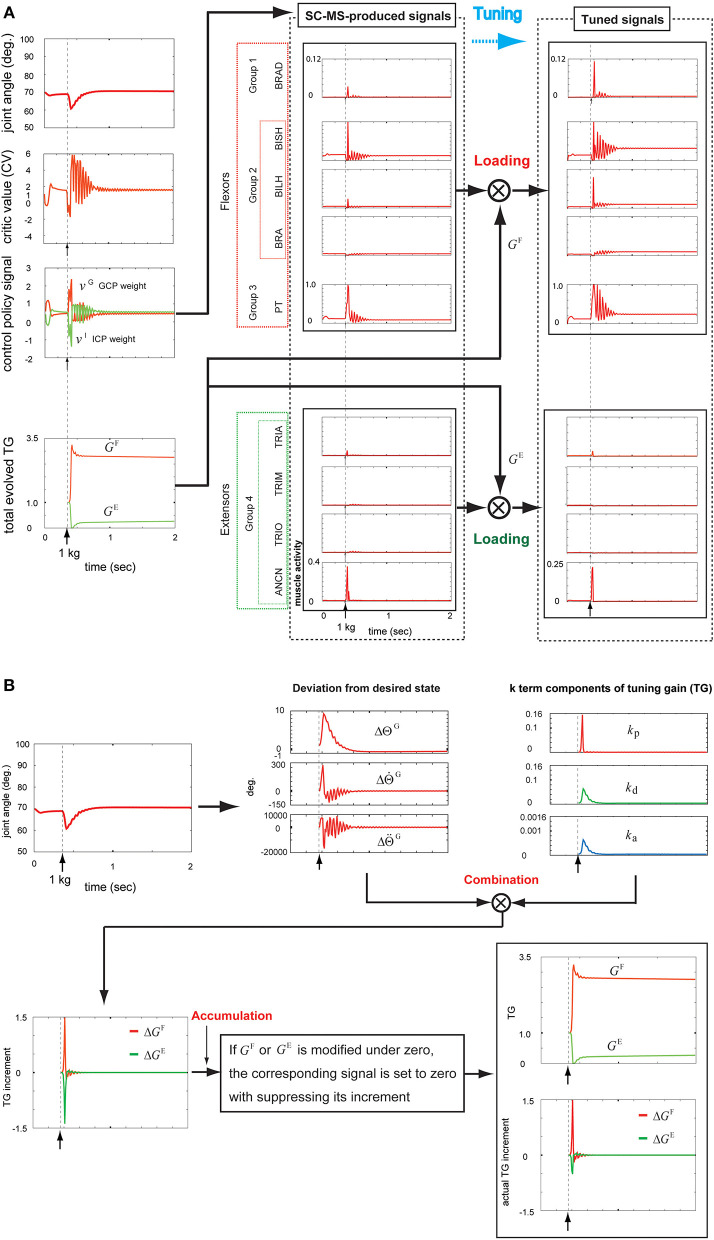
Tuning the learned synergy strategy-based muscle control motor skill (SC-MS) to recruit it after a 1 kg loading. **(A)** Loading the SC-MS-produced signals with tuning gain (TG) signals. **(B)** The process of producing TG signals. The SC-MS produced the activities of eight muscles, which are pronator teres(PT), brachialis(BRA), biceps brachii (long head)(BILH), biceps brachii(short head)(BISH), brachioradialis(BRAD),anconeus(ANCN), triceps brachii(long head)(TRIO), triceps brachii(medial head)(TRIM), and triceps brachii (lateral head)(TRIA).

As shown in Figure 5A, the SC-MS-produced signals were loaded with *G*^F^ and *G*^E^, the agonistic and antagonistic signals of the TG, respectively, according to the recruitment process of a learned MS in Figure 1. These two TG signals were dynamically generated through the following feedback gain process of a learned MS in Figure 5B.

Under the rule based on Equation (5), the three *k*-term components of the TG in the third graph in the top row of Figure 5B were determined according to the joint angle and velocity deviations from the desired states, $\Delta {\Theta_{t}}^{\text{G}}$and $\Delta {{\dot{\Theta }}_{t}}^{\;\text{G}}$, respectively, in the second graph in the top row of Figure 5B. As described in Figure 2A, the component *k*_p_ is designed to be sensitive to the increase in $\Delta {\dot{\Theta }}_{t}^{\;G}$, which is attributed to the high undershooting. Therefore its increase was faster and higher than both *k*_d_ and *k*_a_ that are designed to respond to the increase of $\left||\Delta {\Theta_{t}}^{\;\text{G}}|\right|$, which was attributed to the undershooting or overshooting angle deviation (Figure 2B).

According to Equation (4), these three *k*-term components combined with their corresponding deviations from the desired states to produce three incremental components of *G*^F^ and *G*^E^, as shown in the second and third graphs in the top row of Figure 5B. This combination produced the Δ*G*^F^ and Δ*G*^E^, as shown in the first graph in the bottom row of Figure 5B. According to the rule of Equation (6), *G*^F^ and *G*^E^ must be above zero. Therefore, if either *G*^F^(*t*) or *G*^E^(*t*) is modified to be below zero, the corresponding signal is set to zero by suppressing its increment. Under this rule, Δ*G*^F^ and Δ*G*^E^ were regulated to modify *G*^F^ and *G*^E^, respectively, as shown in the second graph in the bottom row of Figure 5B. The bottom part of this graph shows Δ*G*^F^ and Δ*G*^E^, which actually contribute to modifying the *G*^F^ and *G*^E^, respectively. The tracks of *G*^F^ or *G*^E^ were as follows.

After a sustained 1 kg loading, *G*^F^ increased from 1.0 to its peak value of 3.3 in response to the drop in the joint position, followed by a decrease in responding to the recovery of the preloading context, and finally remained at a stable value of 2.7 to maintain the preloading context. By contrast, *G*^E^ decreased under the same conditions from 1.0 to the lower value 0.0 in response to the decrease in the joint position before increasing, reflecting the preloading context recovery, and finally reached a stable value of 0.25 to retain the preloading posture. Hence, *G*^F^ increased and *G*^E^ decreased from 1.0 during the recovery of the preloading posture in response to a sustained disturbance. These TG signals, as shown in Figure 5A, contributed to additionally modulating the SC-MS through tuning its signals as follows.

The parameter *G*^F^ is reflected in the increased activities of agonists that were kept at higher values in the recovered preloading context compared with their preloading activities. Conversely, the parameter *G*^E^ decreased the antagonist activities and then maintained them at lower values in the recovered preloading context in comparison with their preloading activities.

The aforementioned results demonstrate that the SC-MS can be robustly recruited for a novel feedback context with additional modulation, which was achieved through tuning its signals.

### T-SC in Further Novel Contexts

As shown in section T-SC in a Novel Sustained Disturbance, we verified that an SC-MS can be robustly recruited by tuning it for a novel sustained 1 kg loading, which did not involve learning of the SC-MS. In this section, we demonstrate the versatility of this recruitment process in further novel contexts.

#### Recruitment in Undershooting Attributed to a Novel Sustained 2 kg Loading

**Figure 6:**
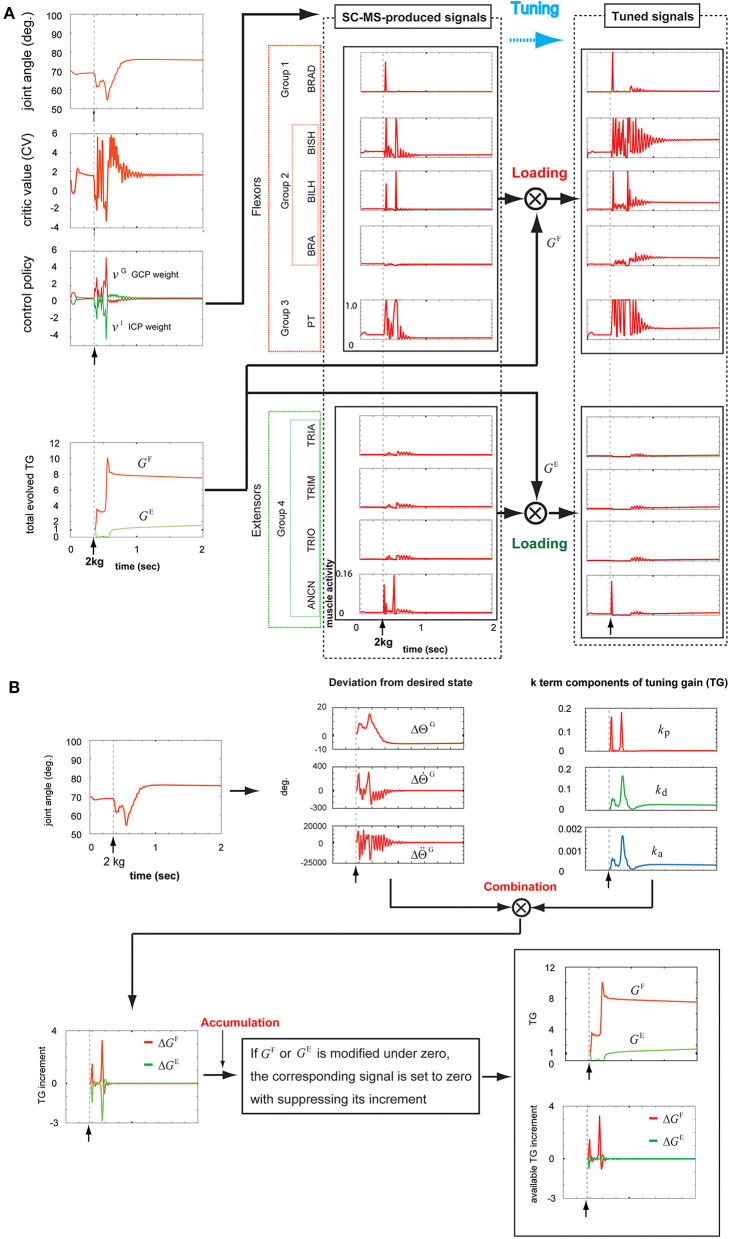
Tuning the learned synergy strategy-based muscle control motor skill (SC-MS) to recruit it after a 2 kg loading. **(A)** Loading the SC-MS-produced signals with tuning gain (TG) signals. **(B)** The process of producing TG signals. The SC-MS produced the activities of eight muscles, which are pronator teres(PT), brachialis(BRA), biceps brachii (long head)(BILH), biceps brachii(short head)(BISH), brachioradialis(BRAD),anconeus(ANCN), triceps brachii(long head)(TRIO), triceps brachii(medial head)(TRIM), and triceps brachii (lateral head)(TRIA).

To examine the recruitment of a learned SC-MS by tuning it in an additional severe undershooting context, we simulated the recruitment of a T-SC-MS for sustained 2 kg loading, which is two times the weight of the 1 kg loading used in section T-SC in a Novel Sustained Disturbance. The simulation of this recruitment process is shown in Figures 6A,B. The top graph of the first column in Figure 6A shows the joint angular trace during the recruitment process of the SC-MS for this disturbance as follows.

After loading with 2 kg, the joint angular trace dropped below the preloading position to about 62° and then increased to be maintained at about 65° for a short time. However, the joint angle declined again to about 55°, but finally recovered to about 75° near the goal state and then was kept stable at that state. Two decreases in angle value and some overshoot during the recruitment of the SC-MS showed an incomplete recovery for 2 kg loading in comparison with the process for 1 kg. This difference is attributed to further severe disturbances over a 1 kg loading. This movement could be achieved through the following recruitment processes of the T-SC-MS.

As mentioned in section T-SC in a Novel Sustained Disturbance, the parameter CV evaluates the recruitment of an SC-MS to achieve the goal state. The second graph of the first column in Figure 6A shows the CV as the evaluation of the recruitment of T-SC-MS for a 2 kg loading. Owing to the aforementioned severe decrease, the CV for a 2 kg loading decreased further than the CV for a 1 kg loading during undershooting. As shown in the third graph of the first column of Figure 6A, this decrease in the CV increased the GCP more than the decrease in the CV under the 1 kg loading. Accordingly, the ICP was suppressed further than that under the 1 kg loading. This CV-driven synergy between two control policies regulates the control redundancy of individual muscle units. Through this regulation, the muscle activities are produced, as shown in the second column of Figure 6A. These signals were loaded with *G*^F^ and *G*^E^ according to the recruitment process of a learned MS in Figure 1. These two TG signals were dynamically produced through the following feedback gain process of a learned MS in Figure 6B.

Under the rule based on Equation (5), the three *k*-term components of the TG shown in the third graph in the top row of Figure 6B were determined by the joint angle and velocity deviations from the desired states, $\Delta {\Theta_{t}}^{\text{G}}$ and $\Delta {{\dot{\Theta }}_{t}}^{\text{G}}$, respectively, in the second graph in the top row of Figure 6B. After loading with 2 kg, as mentioned above, the joint movement developed in two downward steps. In the first step, the component *k*_p_ drastically increased to respond to the increase of $\Delta {{\dot{\Theta }}_{t}}^{\;\text{G}}$, which was attributed to the high-speed downward motion. Concurrently, both *k*_d_ and *k*_a_ increased by less than *k*_p_ because they responded to $\left||\Delta {\Theta_{t}}^{\;\text{G}}|\right|$, the increase in which was less than the increase in $\Delta {{\dot{\Theta }}_{t}}^{\;\text{G}}$. During this step, the response traces of the three *k*-term components were similar to those observed with a 1 kg loading. In the second step, all three *k*-term components increased substantially in response to the large increases in both $\left||\Delta {\Theta_{t}}^{\;\text{G}}|\right|$ and $\Delta {{\dot{\Theta }}_{t}}^{\;\text{G}}$. This is attributed to the feedback context, in which the joint angle state was far from its preloading state with a high downward speed. After this second drop, the joint angular state mostly recovered by 0.8 s to its preloading goal state before slowly reaching the preloading state. In response to this recovery, the *k*_p_ drastically decreased in response to the decrease in $\Delta {{\dot{\Theta }}_{t}}^{\text{G}}$ in downward speed, and both *k*_d_ and *k*_a_ concurrently decreased in response to the decrease in $\left||\Delta {\Theta_{t}}^{\text{G}}|\right|$. After 0.8 s, *k*_d_ and *k*_a_ increased slightly by about 1.0 s in response to the slight overshooting of $\left||\Delta {\Theta_{t}}^{\text{G}}|\right|$ and then decreased quite slowly in response to the quite slow decrease of $\left||\Delta {\Theta_{t}}^{\text{G}}|\right|$ to zero. Under the rule of Equation (4), these three *k*-term components combined with the corresponding deviations from the desired state, which are shown in the second graph in the top row of Figure 6B. These combinations produced the Δ*G*^F^ and Δ*G*^E^, as shown in the first graph in the bottom row of Figure 6B. According to the rule of Equation (6), Δ*G*^F^ and Δ*G*^E^ were regulated to modify *G*^F^ and *G*^E^, respectively, as shown in the second graph in the bottom row of Figure 6B. The bottom part of this graph shows Δ*G*^F^ and Δ*G*^E^, which actually contribute to modifying the *G*^F^ and *G*^E^, respectively. The tracks of the TGs and their contribution to tuning the SC-MS were as follows.

*G*^F^ increased in response to the two drops in joint angle value and then slowly decreased to a stable level. To assist *G*^F^, the parameter *G*^E^ was suppressed during the first drop, but increased substantially during the second drop before slowly dropping to a stable level. These two agonist and antagonist TG signals were loaded onto the SC-MS-produced muscle activities, as shown in the second and third columns of Figure 6A. Through this loading, they were tuned to the optimal muscle activities for recruiting the SC-MS under a 2 kg loading.

#### Recruitment in Overshooting Attributed to a Novel Sustained−1 kg Loading

The overshooting during the SC-MS learning process is transiently driven by incorrectly controlling the joint and is eventually suppressed by gravity. Therefore, the SC-MS learned to control the overshooting with very little extensor activation, which functions as the agonist for overshooting. Because of this learning condition of the SC-MS, the overshooting driven by the sustained negative disturbance on the hand is further severe novel disturbance in recruiting the SC-MS than the undershooting driven by the sustained positive disturbances such as 1 kg or 2 kg loading. Therefore, to recruit the SC-MS during the sustained negative disturbance-driven overshooting, the SC-MS needs to be tuned more than the sustained positive disturbance-driven undershooting. By simulating the recruitment of the T-SC-MS during the overshooting driven by a sustained negative disturbance, we tested the tuning process to robustly recruit the SC-MS in an entirely novel context as follows.

**Figure 7:**
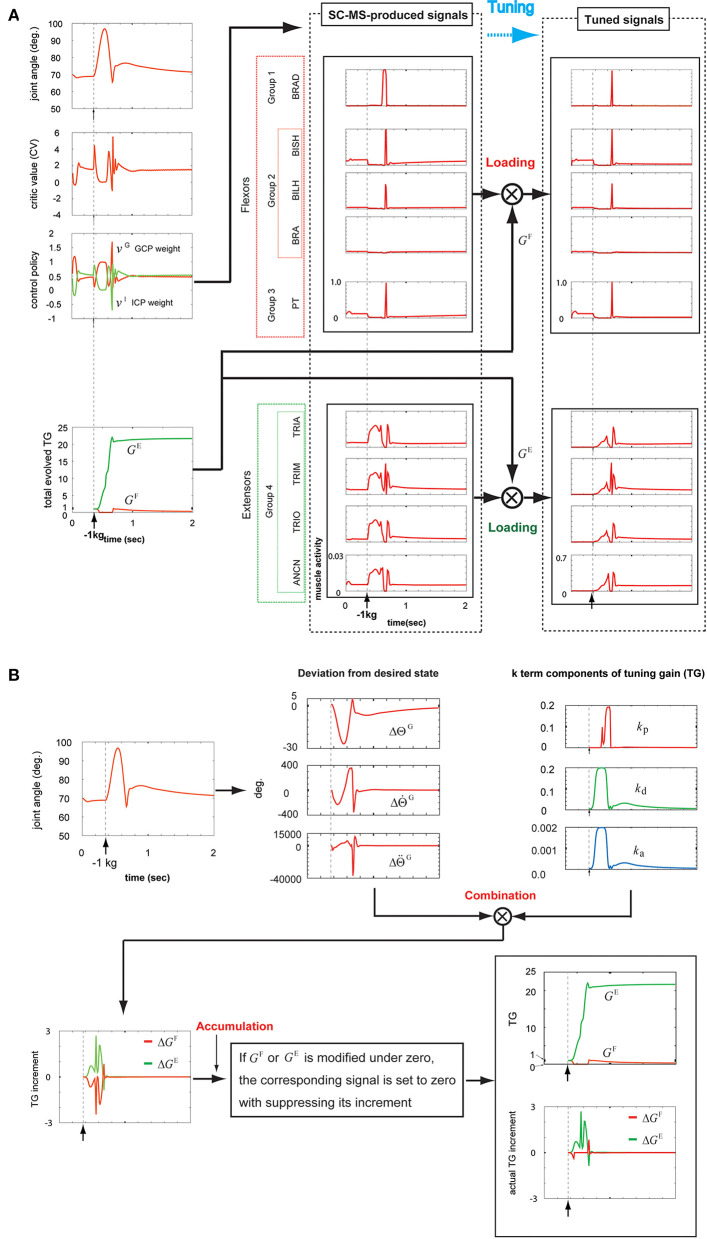
Tuning the learned synergy strategy-based muscle control motor skill (SC-MS) to recruit it after a −1 kg loading. **(A)** Loading the SC-MS-produced signals with tuning gain (TG) signals. **(B)** The process of producing TG signals. The SC-MS produced the activities of eight muscles, which are pronator teres(PT), brachialis(BRA), biceps brachii (long head)(BILH), biceps brachii(short head)(BISH), brachioradialis(BRAD),anconeus(ANCN), triceps brachii(long head)(TRIO), triceps brachii(medial head)(TRIM), and triceps brachii (lateral head)(TRIA).

After loading a −1 kg weight on the simulated hand, the joint angular trace was raised to about 98° and then decreased to about 66° (top graph, first column of Figure 7A). Finally, the joint angular trace overshot by about 76° and then stably recovered to the preloading state. According to this contextual joint angular trace, the CV was determined as shown in the second graph of the first column of Figure 7A. Further, gravity, which reflects the movement, needs to be considered in determining the CV. As mentioned in the first paragraph of this section, it is comparatively easy for an SC-MS to suppress the incorrect control-driven transient overshooting because of gravity during its learning process. Therefore, even if the overshooting attributed to the sustained negative disturbance on the hand is a further severe context for the SC-MS, the CV for recruiting the SC-MS during the overshooting is less than that during the undershooting [second graph, second column of Figure 4 (first column of 5A), and first column of 6A]. Because of this evaluation of the CV, as shown in the third graph in the first column of Figure 7A, the change in the GCP and ICP weights in response to this negative sustained disturbance was suppressed to a small range in comparison with its response to positive sustained disturbances such as a 1 or 2 kg loading. This process was regulated using Equation (1). As mentioned above, this response is attributed to the learning condition of SC-MS, in which the transient overshooting driven by incorrect control is controlled by a small amount of activity of the extensors because gravity contributes to the recovery of the preloading state from its overshooting state. Because of this learning condition of SC-MS, the extensors functioning as agonists for negative sustained disturbance need to be loaded more than the flexors functioning as agonists for positive sustained disturbance. To process this additional modulation, the SC-MS-produced muscle activities shown in the second column of Figure 7A were loaded with the antagonistic and agonistic TGs, *G*^F^ and *G*^E^, respectively. These TGs were produced by the following process.

As shown in the first graph in the bottom row of Figure 7B, the three incremental components of *G*^F^ and *G*^E^ were produced by combining the three *k*-term components of the TG (third graph, top row of Figure 7B) and their corresponding deviations from the desired state (second graph, top row of Figure 7B). These three components of *G*^F^ and *G*^E^ were summed to produce Δ*G*^F^ and Δ*G*^E^, respectively, which were accumulated to produce *G*^F^ and *G*^E^ as shown in the second graph in the bottom row of Figure 7B. The precise process was achieved as follows.

In agreement with the rules shown in Figures 2A,B, during the initial overshooting shown in the first graph in the top row of Figure 7B, the *k*_p_ component was almost suppressed in response to the high overshooting but the *k*_d_ and *k*_a_ components drastically increased in response to the large increase in $\left||\Delta {\Theta_{t}}^{\text{G}}|\right|$. After the joint movement started to recover to its preloading state at about 0.57 s, *k*_p_ drastically increased in response to the large increase in $\Delta {{\dot{\Theta }}_{t}}^{\text{G}}$; this increase was attributed to the high-speed downward motion, and both the *k*_d_ and *k*_a_ components decreased in response to the decrease in $\left||\Delta {\Theta_{t}}^{\text{G}}|\right|$. Thereafter, the joint angle finally recovered to its preloading state via the slight undershooting and the second overshooting, which was less pronounced. In response to this recovery, *k*_p_ decreased to almost zero, whereas *k*_d_ and *k*_a_ stably decreased to their preloading values via their transient increase, as shown in the third graph in the top row of Figure 7B. According to Equation (4), these three *k*-term components combined with their corresponding deviations of desired states to produce the Δ*G*^F^ and Δ*G*^E^, as shown in the first graph in the bottom row of Figure 7B. Under the rule of Equation (6), the Δ*G*^F^ and Δ*G*^E^ were regulated to modify the *G*^F^ and *G*^E^, respectively, as shown in the second graph in the bottom row of Figure 7B. The bottom part of this graph shows the Δ*G*^F^ and Δ*G*^E^, which actually contribute to modifying the *G*^F^ and *G*^E^, respectively. The tracks of the TGs and their contribution to tuning the SC-MS were as follows.

*G*^E^ substantially increased during the overshooting and then stabilized at a lower level owing to the recovery of the preloading state. To assist *G*^E^, *G*^F^ was completely suppressed during the overshoot and then increased for a very short time before decreasing slowly to a stable level.

Because of this modulation of *G*^F^, as shown in the top of the second and third columns of Figure 7A, the SC-MS-produced signals of flexors were dynamically unloaded in response to the overshooting attributed to a negative sustained disturbance because they were antagonists for the negative disturbance. In comparison with the flexors, the extensors were highly loaded with *G*^E^ to function as agonists against the overshooting (the bottom part of the second and third columns, Figure 7A). Through this tuning process, the handicap in recruiting an SC-MS under overshooting conditions attributed to sustained negative disturbances, which was mentioned in the first paragraph of this section, could be overcome.

## Discussion

A learned MS can potentially be used for effective motor control in a novel context. In addressing this issue, we hypothesized that an MS can be retained through learning it in the CNS and then recruiting it. Through the simulation using the proposed neurophysiological computational model, we have shown that the MS might be retained through learning the muscle synergy to achieve its task and recruited through dynamically tuning it according to novel feedback contexts. In this tuning, the learned muscle synergy, termed SC-MS, produces the muscle control signals through its dynamic modulation according to the feedback context and these signals are additionally loaded with tuning signals, termed TG signals, which are dynamically modulated according to the feedback context. Through this dynamic modulation, a skilled MS might be recruited in a variety of conditions besides those experienced during motor learning. Furthermore, this involvement of the muscles' synergy with a skilled MS demonstrates that it might subserve the learning and retaining of an MS in the CNS.

### Dynamic Modulation of an MS According to the Feedback Context

To recruit a learned MS for novel contexts, we assumed that a learned MS is dynamically modulated for the feedback context. In addressing this issue, we used the concept of SC (Min et al., 2018), which dynamically regulates the redundant functional roles of individual muscles according to consecutive feedback contexts. As described in Equations (1) and (2), this SC-driven regulation contributes to the dynamic modulation of an MS for the feedback context. Consequently, this modulation contributes to robust recruitment of an MS in various novel feedback contexts that did not involve the learning of an MS, as shown in Figures 4, 5A, 6A, 7A. These results show that the SC may be an optimal strategy to learn an MS and to recruit it.

### Robust Recruitment of a Learned MS Through Tuning It According to the Feedback Context and Its Implications

Even if an SC-MS is modulated according to the feedback context as mentioned in the above subsection, this modulation is learned in the dynamics under the learning context of SC-MS. Because of this learning condition, to robustly recruit an SC-MS in a novel context, it needs to be additionally tuned. To validate this tuning, we hypothesized that a muscle loading tuner may operate in the CNS to tune the SC-MS through dynamically loading its muscle control signals according to the feedback context. This hypothesis was validated with the simulation results shown in Figures 5A, 6A, 7A, in which the SC-MS could be successively recruited through dynamically loading its muscle control signals according to the feedback context under three different novel sustained disturbances. This recruitment may involve the rapid adaptation of motion control to novel contexts without learning a new MS for them. If this rapid adaptation is impaired, the normal motion control in novel dynamic contexts may be seriously disturbed. To test the potential clinical implications, this hypothesis needs to be further studied in neurophysiology. Through this study, the proposed model may provide a new clinical view of motion control disorders attributed to cortico-BG loop-related CNS diseases in pathophysiology and therapeutics/rehabilitation. Furthermore, through the transcortical circuit, the recruitment-produced muscle control signals may be transferred to the cerebellum as a correction signal to train a neural network, on which a feedforward motor command is generated in the cerebellum (Kawato et al., 1987; Kawato, 1990; Kambara et al., 2009). Therefore, the T-SC may involve robust feedforward motion control in novel contexts.

Previous studies, such as proportional integral derivative control (Petkos and Vijayakunar, 2007) and optimal feedback control (Todorov and Jordan, 2002; Liu and Todorov, 2007) in modeling the feedback control process, focused only on the correction of the motor control error but did not address the contribution of a learned MS to feedback control. Our new approach to recruitment of a learned MS in novel contexts may offer a new viewpoint for this previously unaddressed feedback control issue.

### Recruiting a Learned MS via the Cortico-Basal Ganglia Loop

The BG contributes to “stabilization augmentation” by facilitating an optimal activity that fits the desired situation and context while suppressing other ongoing CNS activities that would interfere with the desired behavior (Mink, 1996). Furthermore, Turner and Anderson (1997) showed that movement-related changes in pallidal discharge to specific parameters of movement are discharge of neurons in the skeletomotor portions of both pallidal segmentations. This BG response is demonstrated by encoding the combination of the sensory and contextual state through the sensory feedback process, which may involve online motion control with the selective facilitation and suppression of different frontal thalamocortical circuits (Turner and Anderson, 1997). As the BG reinforces a new MS through reinforcement learning and retains it subsequently (Lehéricy et al., 2005), this online motion control role of the BG may involve the recruitment of a learned MS retained in the BG, which is dynamically modulated by the selective facilitation and suppression of different frontal thalamocortical circuits. Based on the aforementioned previous studies, this cortico-BG scheme may be a common framework for the learning and recruitment of an MS in the CNS. Therefore, the T-SC-driven recruitment of a learned MS though the cortico-BG loop may involve different kinds of motion control, which need to respond to various sensory feedback contexts via the M1 from different sensory areas, including the somatosensory cortex and the visual cortex. This hypothesis may be reasonable, even if it has recently been demonstrated that the roles of neural structures differ between different tasks (Paparella et al., 2020).

### Muscle Control Scheme of the Corticospinal Tract in Recruiting an SC-MS

The experimental evidence introduced in section Recruiting a Learned MS via the Cortico-Basal Ganglia Loop shows that the BG may retain a learned MS and involve the recruitment of it to control movement according to the feedback context. Based on this concept, to recruit an SC-MS according to the feedback context, we assumed that the BG may dynamically modulate an SC-MS with the synergistic combination of two control policies of the SC, GCP, and ICP, which is driven by a combination of their inhibition and disinhibition. As shown in Figure 1, this synergistic combination of GCP and ICP in the BG produces muscle control signals through the corticospinal tract. As outlined in section Introduction, GCP-driven signals may function as group unit control signals that are decoded into synergistic combinations of MPs (Bizzi et al., 1991; d'Avella et al., 2003) retained in the spinal cord because the group units produce the contraction sets of muscles termed MPs in processing the SC. Furthermore, as the ICP-driven signals serve as the control signals for individual muscle units, they may be directly copied from the corticomotor neurons among the CSTs to MNs. Therefore, the ICP-driven signals sculpt GCP-driven signals through their synergistic combination to optimally modulate an SC-MS according to the feedback context. This recruitment of an SC-MS may support the concept introduced in section Introduction that muscle activities are produced by combining two pathways of MNs (Rathelot and Strick, 2009).

### Evaluating the Proposed Model in Comparison With Human Subjects

Evaluating the proposed computational model in comparison with human subjects, the two disadvantages of the computational model were as follows.

In this study, an SC-MS was learned only through one learning experience of a particular task, which was to move the hand to its goal within a limited joint angular range as described in section Learning and Recruitment Condition of an SC-MS. While learning the SC-MS, no disturbances were involved (Min et al., 2018), as described in section Learning and Recruitment Condition of an SC-MS. Therefore, the recruitment of an SC-MS under sustained disturbance was simulated as a pure novel recruitment, as described in section Results. To evaluate this simulation in comparison with human subjects, as shown in the top row of Figure 4, we approximated a pure novel recruitment as closely as possible using only those data that were recorded during the first trial for each of the four subjects. However, the subjects have experienced and learned various tasks during their whole life and thereby have experienced various tasks under various sustained disturbances. Therefore, the sustained 1 kg loading on the hand is not a pure novel context for these subjects. Consequently, this should be taken into account when evaluating the simulation results through a comparison with the subjects' movements. Because of the disadvantage attributed to pure novel recruitment, an SC-MS is even more difficult to recruit under novel sustained disturbances, such as a sustained 1 kg loading, than the subjects. Considering this disadvantage, we may evaluate that an SC-MS can be robustly recruited through the proposed recruitment model termed T-SC.

As mentioned in section Introduction, innate and learned MSs are recruited in the CNS for effective and fast motion control in response to novel external disturbances. To validate this, the recruitment of a learned MS in a pure feedback control process is the most optimal task because the pure feedback control, which is not involved in the prediction of any disturbance, may need the most effective and fast response to the feedback context. Therefore, as described in section Results, novel recruitment with T-SC was simulated in pure feedback control. To evaluate this simulation by comparison with human subjects' movements, as shown in the top row of Figure 4, we approximated this pure feedback control process as closely as possible, as described in section Experimental Setup, through an experimental setting in which the subjects were blindfolded and not informed regarding the timing of the loading. To avoid the weight being misloaded on the subjects' hands, as shown in Figure 3B, the distance between the initial falling point of the weight and the initial position of the hand was set close to zero. Further, we instructed the subjects not to predict the timing of the loading weight. However, even though this instruction was given to the subjects, they might instinctively have some preliminary joint stiffness by co-contraction of both agonists and antagonists in preparation for the incoming disturbance before loading. Because of this, as shown in the top row of the right column of Figure 4, the mean joint angular trace of the subjects after loading undershot was slower than the simulating joint angular trace. In evaluating the simulation results in the top row of Figure 4, we considered that the simulation model was disadvantaged in responding to a disturbance in comparison with human subjects.

## Data Availability Statement

All datasets generated for this study are included in the article/supplementary material.

## Ethics Statement

All subjects were provided written informed consent prior to their participation. The protocol was approved by the ethics committees of the Tokyo Metropolitan Institute of Medical Science, and it was conducted in accordance with the ethical standards of the Declaration of Helsinki.

## Author Contributions

KM conceived and designed the theoretical and computational model and the experiments. KM and JL conducted the experiments. KM and SK analyzed the data. KM, SK, and JL wrote the manuscript. All authors read and approved the version to be published and agreed to be accountable for all aspects of the work.

## Conflict of Interest

The authors declare that the research was conducted in the absence of any commercial or financial relationships that could be construed as a potential conflict of interest.
